# Analysis of the drivers of ASF introduction into the officially approved pig compartments in South Africa and implications for the revision of biosecurity standards

**DOI:** 10.1186/s40813-022-00286-7

**Published:** 2022-10-06

**Authors:** Carla Stoffel, Patrik Buholzer, Angela Fanelli, Marco De Nardi

**Affiliations:** 1 SAFOSO, Waldeggstrasse 1, 3097 Liebefeld, Switzerland; 2 Department of Veterinary Medicine, University of Bari, Valenzano, Bari, Italy

**Keywords:** African Swine Fever, South Africa, Compartmentalization, Risk assessment, Expert elicitation, Farm biosecurity, Pork industry, Business continuity, Capacity development

## Abstract

**Background:**

While African Swine Fever (ASF) virus has historically circulated in wild pigs and in *Ornithodoros* ticks in parts of South Africa, the virus has spread among domestic pigs throughout the country since 2019. South Africa’s compartment system has been used as a mainstay approach to protecting the swine industry in the face of ASF. However, in 2020, two compartments broke down with ASF. The objectives of this study are to investigate the drivers for ASF introduction into the compartments, to categorize compartments by risk of ASF introduction, and to make corresponding recommendations. The relevance of risk factors for ASF introduction for each compartment were investigated among veterinarians and farm managers. The analysis of risk factors weighted according to an expert elicitation were used to categorize compartments into risk levels.

**Results:**

Drivers of disease related to human behaviors and to domestic pig management are perceived by farm managers and veterinarians of the compartments to be critical for ASF introduction into compartments in South Africa. Twenty-four units were categorized as high risk, forty-seven as medium risk, and twenty-four as low risk. “Insufficient boot and clothing biosecurity by animal health personnel” was identified as a relevant risk factor in all high risk units. Other prominent risk factors were “insufficient boot and clothing biosecurity by external people,” “underreporting of suspect ASF cases,” “improper hunting/ culling of wild suids inside the compartment,” “un-tested introductions into the herd,” and “entry and contact with free-roaming pigs.” The roles of wild pigs and competent vectors are considered minimal. There is a need for revision of the compartment standards and training of compartment personnel on the standards. The major gaps identified in the standards were absence of a monitoring programme to assess biosecurity implementation and suboptimal surveillance testing and audit strategies.

**Conclusions:**

The results of our study confirm that ASF is increasingly an anthropogenic problem. Updating the compartment standards and addressing gaps in the knowledge of compartment personnel on ASF are most critical. To enhance compliance with biosecurity measures and thus control the disease, close engagement with all stakeholders linked to the compartments is needed.

**Supplementary Information:**

The online version contains supplementary material available at 10.1186/s40813-022-00286-7.

## Background

### ASF situation in South Africa

African Swine Fever (ASF) is a major threat and challenge for pig industries globally. While ASF virus circulates in wild pigs and in *Ornithodoros* ticks in parts of South Africa [4], there was a resurgence of ASF in domestic pigs outside the control zone starting in Gauteng Province in 2019 [1]. Since this incursion through 2021, ASF spread to domestic pigs in five other provinces. In 2020, three units of two official pig compartments broke down with ASF.

**Fig. 1:**
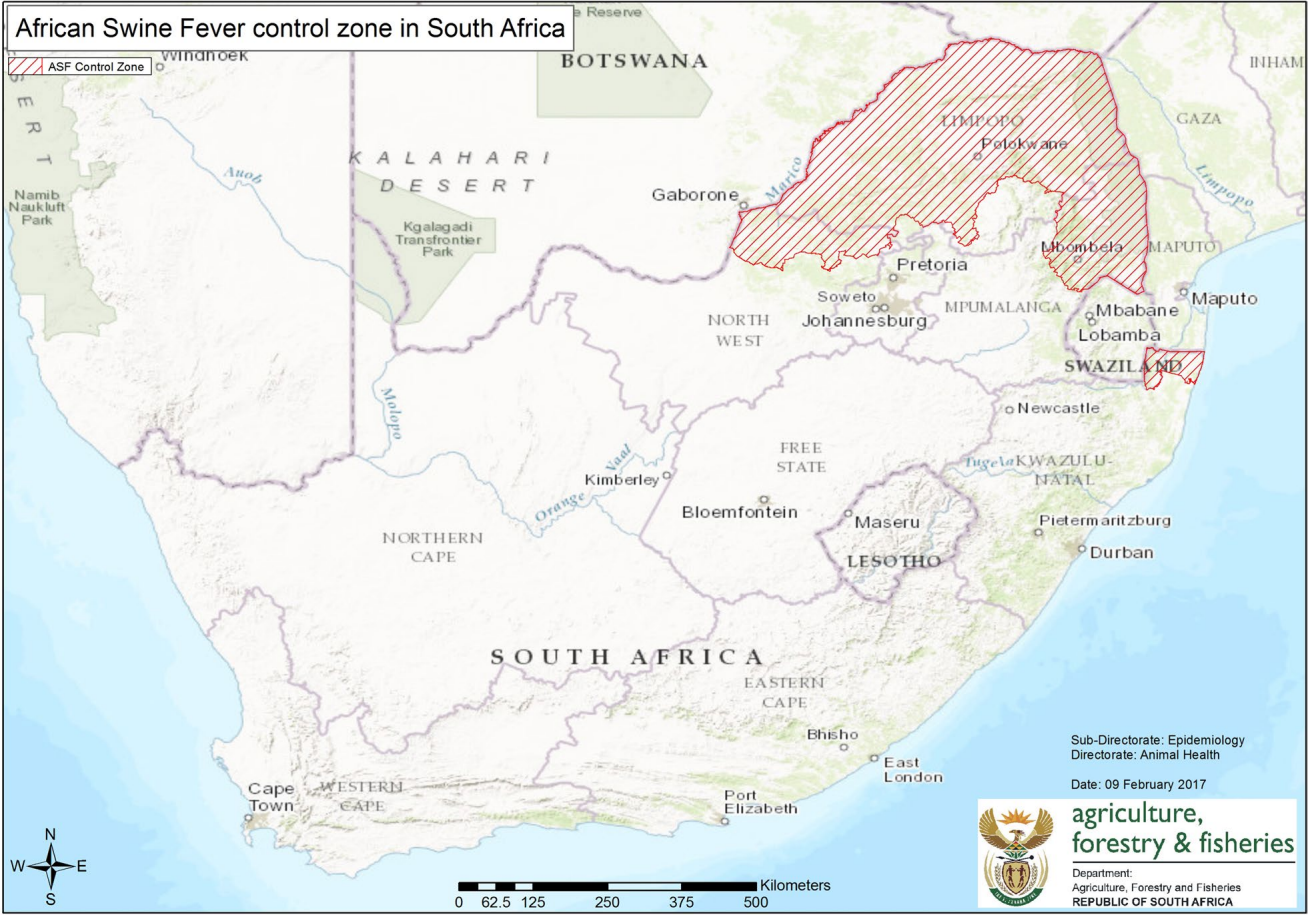
The ASF Control zone in the northeast part of South Africa is marked in red [7]. A resurgence of ASF started in Gauteng province just south of the ASF control zone since 2019 and continued to spread. ASF-affected pig compartments are located in Gauteng province and the North West province

ASF is endemic to a designated part of South Africa known as the ASF control zone established in 1935 ([25], Fig. 1). In the ASF-endemic area, pig-proof pens and double-fencing are used to prevent contact between domestic and wild pigs [1]. Movement permits issued by a state veterinarian are required for the movement of domestic or wild pigs and their products within, from, and into the control zone [12].

Over the past 6 decades, warthogs have been widely translocated from the north to the south of the country where they have become an invasive species [4]. *Ornithodoros* ticks and ASF virus have also been found in warthog burrows outside the control zone [4]. Although ASF outbreaks in domestic pigs outside the control zone have not been directly linked to the sylvatic circulation of ASF virus in warthogs and *Ornithodoros* ticks, the continued expansion of warthog populations and possible utilization without proper biosecurity measures is a possible threat to ASF introduction to domestic farms. The threat is greater for small-scale pig farmers than for commercial producers that apply strict compartmentalization protocols [4].

South Africa’s swine industry is dualistic, with about 25% of the pigs in South Africa belonging to emerging or smallholder pig farmers [6]. In contrast to commercial pig farming, emerging pig farming is characterized by relatively small enterprises with poor management and poor biosecurity. This dualism is a major determinant in the spread of ASF virus, as early disease detection and control is challenging in the small-scale sector due to lack of resources. While this study is limited to the compartment system, further areas of research could include expansion of the analysis to the entire swine industry.

All outbreaks of ASF in domestic pigs outside the control zone between 2012 and 2021 can be linked to human behavior [20, 32]. The outbreak in Gauteng province in 2012 was traced to the illegal movement of sick pigs from the control zone to a sales yard in Mpumalanga province [13]. Most of the outbreaks in 2016 and 2017 were linked to illegal movement of pigs and pork and to swill feeding [20].

In early 2020, ASF outbreak events were reported in domestic pigs in the provinces of Free State, Mpumalanga, and Eastern Cape before spreading to Gauteng [1]. It is suspected that trade of pigs and pig products between the provinces of Gauteng, Mpumalanga, Free State, and North West compounded the risk of ASF spread [1]. Outbreaks in the Western Cape in 2021 were possibly linked to the settlements populated by immigrants from the Eastern Cape [32].

A compartment broke down with ASF for the first time in November 2020 in Gauteng province. Since then, only one other compartment has become affected by ASF in the North West province in March 2021. The routes of ASF introduction for either outbreak have been speculated but not proven.

**Fig. 2:**
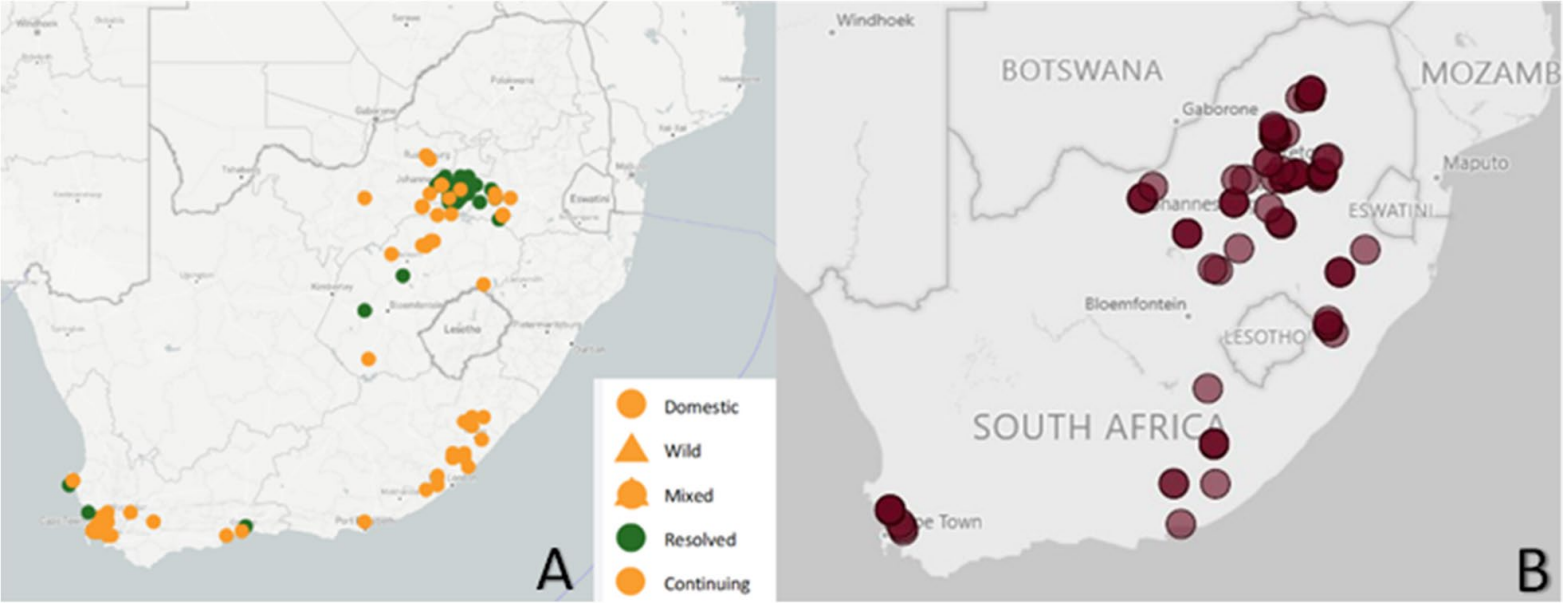
Reported ASF outbreaks in domestic pigs in South Africa in 2021 (**A**) [31]. ASF spread from Gauteng province (northeast) near the ASF-control zone, reaching the Western Cape (southwest) and Eastern Cape (southeast) provinces. The WOAH bears no responsibility for the integrity or accuracy of the data contained herein, in particular due, but not limited to, any deletion, manipulation, or reformatting of data that may have occurred beyond its control. Locations of all ASF compartments in South Africa (**B**) (South African Pork Producers’ Organisation (SAPPO). Pig compartments. n.d. Unpublished). Compartments are clustered in the northeast of the country, with most compartments in Limpopo, Gauteng, and Mpumalanga provinces. Many compartments are in or near the ASF control zone where ASF is endemic in wild pigs

Six of South Africa’s nine provinces had continuing outbreaks of ASF in domestic pigs in 2021 (Fig. 2A). One hundred and forty-five outbreaks (145) were reported in the country to the World Organisation for Animal Health (WOAH) in 2021, which is five times as many as in the previous year (WOAH) [31]. According to the South African Pork Producers’ Organisation (SAPPO), the alarming increase puts commercial pig farmers at great risk, including ASF compartments [27]. Domestic pigs in KwaZulu-Natal, the Northern Cape, Limpopo remain ASF-free at this time.

### Compartment organization

Compartmentalization for pig units began to develop in South Africa during the 1950s due to the endemicity of ASF in wild pigs in the north of the country. Compartmentalization protocols were formalized and officially published from 2001 as voluntary systems [26].

The compartment system in South Africa is administered and controlled by the Department of Agriculture Land Reform and Rural development. A standard document issued by the Department of Agriculture, Forestry and Fisheries (DAFF) describes procedures for registration of and minimum standards for a veterinary approved pig compartment [5]. The compartment system is promoted by SAPPO to farmers, abattoirs and potential importing countries of South African pork and has therefore become an industry standard.

In these governmental compartment standards, a pig compartment is defined as a physically defined establishment surrounded by a physical barrier (i.e., fence) where a pig population is contained under a defined biosecurity management system with a distinct health status with respect to specific diseases for which mandatory surveillance, control and biosecurity measures have been applied [5].

There are seventy-five (75) compartments, each composed of one to eight units, for a total of one hundred twenty-seven (127) units across eight of South Africa’s nine provinces. Each compartment has a unique identifying compartment registration number managed by SAPPO. Each unit is typically one type of swine production enterprise, such as a farrow-to-finish facility or an artificial insemination station.

Since the compartments are under different ownership, they are potentially operated under different management systems. While all compartments are required to implement the standards outlined in the official government standards, some compartments are also members of the Pork360 accreditation scheme. Pork360 is a farm assurance system developed by SAPPO to address best practices and processes that comply with global standards to ensure safe, fresh and affordable pork throughout the pork value chain [34]. Considering that each compartment that shares the same ownership is likely operated under the same if not similar management systems, there are approximately fifty-seven (57) different owners and thus possibly up to fifty-seven (57) different management systems.

Compartments are clustered in the North east of the country, with most compartments in Limpopo, Gauteng, and Mpumalanga provinces (Fig. 2B). Many compartments are in or near the ASF control zone where ASF is endemic in wild pigs and where the virus is found in *Ornithodoros* ticks.

Each compartment unit is assumed to have a unique farm manager that oversees the whole compartment including compartment personnel that are in contact with pigs. The thirteen (13) private consulting veterinarians for the units in the different provinces report to approximately thirty-nine (39) state veterinarians. Private consulting veterinarians play an important role in ensuring the proper implementation of the standards through their visits to the units once every 2 months (A. Calitz, SAPPO, personal communication). The consulting veterinarians are assumed to have first-hand knowledge of the compartment’s management practices and swine health status. State veterinarians, on the other hand, are responsible for the compartment audits every two years.

### Relevance of this study

Freedom from ASF can be demonstrated in a compartment even when ASF is known or is assumed to be present in the country. A well-designed and implemented compartment is meant to prevent the introduction of ASF and thereby maintain business continuity including exports in the face of ASF outbreaks in the country. Since ASF compartments are held to specific standards to maintain the biosecurity barrier, identifying the possible gaps in biosecurity that have allowed for and may lead to the introduction of ASF into compartments is of critical importance to the swine industry of South Africa. Swine industries in other countries can also learn from and address these gaps in their own compartmentalization designs. Once the gaps have been identified, they can be prioritized and addressed in a holistic, systematic, and institutionalized approach for all compartments.

### Objectives of the study

The objectives of the study presented here are to investigate the drivers for ASF introduction into compartments in South Africa, to categorize compartment units by risk of ASF virus introduction relative to each other, and to make corresponding recommendations for prevention of ASF introduction into compartments based on the identified critical risk factors and risk factor categories.

## Results

### Online questionnaire

#### Demographics

All one hundred twenty-seven (127) compartment units were covered by the questionnaire between the responding farm managers and the veterinarians, including those that had been affected by ASF to date of publication. Eighty-six (86) of ninety-seven farm (97) managers responded (89% response rate) covering one hundred and one (101) units, and all thirteen (13) consulting veterinarians responded (100% response rate) covering one hundred eighteen (118) units. About 84% of farm manager respondents were male, while 61.5% of consulting veterinarian respondents were male.

In the following sub-chapters (“Compartment characteristics”–“Drivers of ASF introduction” sections), the unweighted results only of the farm managers are mostly reported here, since farm managers are more closely involved with day-to-day operations of the compartment as compared to the veterinarians. However, some questions were only included in the questionnaire for veterinarians (“Internal surveillance system” section). Notable differences between the responses of the two groups are highlighted at the end of the “Inconsistencies between veterinarian and farm manager responses” section.

#### Compartment characteristics

The most frequently represented facility type was farrow-to-finish (46.5% of units) followed by weaner-to-grower (16.8% of units). Most units would be considered large based on the number of animals housed. Nearly three quarters of grower facilities reported that they house over 1,500 growers (71.3%), and around two thirds of weaner facilities reported housing over 1,500 weaners (67.3%). Almost all units (95%) had indoor only housing, while 5% of units have mixed indoor and outdoor housing. The number of personnel in contact with pigs on a daily basis was reported as greater than seven (7) for 77% of units. The appropriate numbers and types of personnel for a farm are important to guarantee compliance of all personnel with biosecurity measures.

#### Biosecurity

All compartment units were reported to have an internal biosecurity plan that is approved by the Veterinary Authority and that takes into consideration the minimum standards for a veterinary approved pig compartment as per the governmental standards [5]. Over three quarters of units were also members of the Pork360 accreditation scheme (78%). Around 40% of units were reported to apply biosecurity standards in addition to the governmental standards and Pork360 (41.6%). Dry showers, vehicle access restriction, vehicle & boot disinfection, visitor restrictions, animal quarantine protocols, and additional trenches and fences were reported in some units.

For further questions on biosecurity, respondents were given the answer choices of “agree,” “partially agree,” “disagree,” or “I don’t know/ I am not sure.” All units were reported to have a monitoring programme to assess the implementation of biosecurity measures according to standard operating procedures (SOPs). A contingency plan in case of ASF occurrence is reportedly in place for nearly two thirds of units (60.4%). The main barrier to developing a contingency plan was reported to be lack of dedicated finances to control an ASF outbreak.

Farm managers agreed that the internal auditing mechanism includes regular review and updating of biosecurity measures and is designed to identify breaches in biosecurity measures for almost all units (96%).

#### Active farm surveillance

Even though active testing for ASF is a requirement of the governmental standards [5], some compartment units reported not carrying out active testing for neither ASF nor other transboundary animal diseases (4% of units). Three (3) units were reported as having lab-confirmed positive ASF tests in the last 2 years.

#### ASF knowledge and education efforts

Most of the questions in this section were included only in the questionnaire for farm managers. For over 75% of units, farm managers reported that they have had training on pig diseases in the last 5 years (87.1%) that they have had specific training on ASF in the last 5 years (77.2%), and that compartment personnel that are in contact with pigs have had training on ASF in the last 5 years (75.2%). Out of “basic,” “medium,” or “good” answer options, farm managers rated their overall knowledge on ASF modes of transmission and clinical signs as “good” for half of the units and rated their overall knowledge of ASF prevention and control strategies as “good” for over two thirds of units (68.3%).

The capacity of those compartment personnel who are in contact with pigs to recognize ASF clinical signs was rated as “medium” for nearly half of units (46.5%). Compartment personnel who are in contact with pigs were rated as having “basic” capacity to recognize ASF clinical signs for 41.6% of units.

#### Internal surveillance system

Only veterinarians were asked questions about the internal surveillance systems of compartments. The response options were, “unlikely,” “likely,” “very likely,” or “I don’t know/ I am not sure.” Veterinarians reported that it is very likely that the compartment personnel of the (group of) compartment(s) that are in contact with pigs report animals potentially affected by ASF to their managers for 57.6% of compartment units. Veterinarians also reported that it is unlikely that the compartment personnel report animals potentially affected by ASF to their managers for 8.5% of units.

It was reported very likely that the Veterinary Authority is notified in the event of a suspect ASF case for nearly all units (97.5%). Serological testing has been implemented at least every six months in 2020 and 2021 as per the governmental standards for nearly all units (99.2%). For one compartment unit, serological testing has not been implemented at least every 6 months in 2020 and 2021. Serology or virus identification for ASF in addition to the testing required by the government standards in 2020 and 2021 have not been done for nearly all units (95.8%). Out of “low,” “medium,” or “high” answer options, the capacity of the surveillance system to detect an occurrence of ASF is reported as “low” for five (5) units (4.2%). The median rating, however, was that the surveillance system had “high” capacity.

#### Drivers of ASF introduction

Thirty-four (34) risk factors relevant to the introduction of ASF into a compartment were identified and classified into in five categories: ‘domestic pigs’, ‘human behaviors and activities’, ‘wild suids’, ‘competent vectors’, and ‘fomites.’ The risk factors were presented to the respondents to rate the relevance of factors towards the risk of ASF introduction into the compartment unit(s) under their responsibility. Risk factors are referred to here by their proxy names, which can be found in Table 1. The complete risk factor descriptions can be found in Additional file 1. For this section of the questionnaire, respondents were asked to rate their perception of the relevance of each risk factor as “negligible,” “low,” “medium,” “high,” or “not applicable.” Four of the five categories were represented in the top ten ranked risk factors for farm manager and veterinarians. Only the ‘wild suids’ category was not represented among the top ten risk factors for both groups.

**Table 1:** Risk factors relevant to ASF introduction into compartments in South Africa by category using proxy names for simplicity

Category	No	Proxy risk factor name
Domestic pigs	1	On-farm pig density
	2	Proximity to farms with poor biosecurity
	3	Proximity to ASF-affected farms
	4	Un-tested introductions into the herd
	5	Use of un-tested breeding boars
	6	Use of uncertified genetic material
	7	Entry of free-roaming pigs
	8	Contact with free-roaming pigs
	9	Return of live pigs
Human behaviors and activities	10	Insufficient boot and clothing biosecurity by external people
	11	Insufficient boot and clothing biosecurity by animal health personnel
	12	Insufficient cleaning & disinfection of boots, clothes, facilities, and equipment
	13	Feeding of food waste
	14	Underreporting of suspect ASF cases
	15	Improper carcass disposal of sick pigs
	16	Improper on-site slaughter
	17	Improper hunting/ culling of wild suids inside the compartment
	18	Improper hunting/ culling of wild suids in proximity to the compartment
	19	Meals outside designated areas
Wild suids	20	Wild suid entry
	21	Contact with wild suids
Competent vectors	22	Tick vectors
	23	Biting flies
Fomites	24	Insufficient decontamination of swine transport vehicles
	25	Insufficient decontamination of non-swine delivery vehicles
	26	Insufficient decontamination of own tractors & lawnmowers
	27	Same-vehicle transport
	28	Abattoir transport
	29	Contaminated feed or bedding
	30	Improper disposal of carcasses and manure
	31	Insufficient control of scavenger animals within the compartment
	32	Insufficient control of scavenger animals in proximity to the compartment
	33	Insufficient pest control
	34	Regular presence of pets

Eight (8) of ten (10) top risk factors are shared between the farm managers and the veterinarians (Additional file 2). For both groups, the highest number of top ten risk factors were in the ‘fomites’ category (4 risk factors), followed by ‘domestic pigs’ (3 risk factors), ‘human behaviors & activities’ (2 risk factors), and ‘competent vector’ (1) categories. Among the top ten risk factors, there was agreement that on-farm pig density, proximity to farms with poor biosecurity, and insufficient control of scavenger animals in proximity to the compartment in particular were perceived as “high” or “medium” risk for the units. Proximity to ASF-affected farms was rated “high” or “medium” for seven (7) out of seventeen (17) units (41%) in Limpopo, the province closest to the ASF control zone.

#### Inconsistencies between veterinarian and farm manager responses

The farm managers and veterinarians responded differently to the questions in this section which are worth noting. Veterinarians reported that many fewer units (21.2%) apply biosecurity standards in addition to the governmental standards and Pork360 as compared to the farm managers (41. 6% of units). Farm managers named specific additional standards that are applied in some compartments including those from DAFF, Sustainable Agriculture in South Africa (SIZA), Number Two Piggeries (N2P), Pick n Pay and Woolworths. Veterinarians disagreed that a contingency plan is in place for two thirds of units, while farm managers reported that 60.4% of units have a contingency plan in place.

Veterinarians reported for more units (36.4%) that the capacity of those compartment personnel who are in contact with pigs to recognize ASF clinical signs was “good” compared to farm managers (11.9% of units). Veterinarians reported for fewer units (22.0%) that the capacity was “basic” compared to the farm managers (41.6% of units).

Although farm managers tended to rate risk factors higher than the veterinarians, the farm managers and the veterinarians mostly agreed on the top “high” and “medium” risk factors. However, the farm managers and veterinarians rated a few risk factors differently. “Underreporting of suspect ASF cases” and “insufficient decontamination of non-swine delivery vehicles” were rated more frequently “high” or “medium” by farm managers than by veterinarians. While “insufficient cleaning & disinfection of boots, clothes, facilities, and equipment” and “insufficient decontamination of own tractors & lawnmowers” were rated more frequently “high” or “medium” by veterinarians than by farm managers. The percentages of compartment units by rating of perceived relevance of all risk factors reported by both groups are shown in Additional file 3.

### Expert elicitation and the categorization of units by risk

Experts weighed the relative importance of the five categories of risk factors to ASF virus introduction into compartments in South Africa out of one hundred (100) points (Table 2). The most important category of risk factors for ASF introduction based on the median score was ‘human behaviors & activities.’

**Table 2:** Expert elicitation results for weighing of categories of risk factors in the context of the swine compartment system in South Africa out of 100 points

Category of risk factors	Median score
Domestic pigs	25
Human behaviors and activities	45
Wild suids	5
Competent vectors	5
Fomites	20

The averages weighted by the uncertainty scores were similar to the average scores for all categories. It was therefore concluded that uncertainty did not play a major role in the elicitation. Furthermore, there was a consensus for 83.6% of the scores at the 60% threshold above and below the median scores, which is satisfactory [28]. The range of scores was mostly reduced for the categories between the first and second round of elicitation, especially for the ‘domestic pigs’ and ‘human behaviors & activities’ categories, indicating a higher consensus between experts in the second round. All eleven (11) experts agreed with the median scores from the second round of elicitation.

Upon applying the weights of the risk categories from the expert elicitation to the risk scores provided by farm managers, the units were distributed into risk groups “low,” “medium,” or “high” by interquartile range (IQR) (Table 3). The IQR is considered the best measure of variability for skewed distributions [3].

**Table 3:** Distribution of units by risk groups and province according to the interquartile range (IQR)

Province	Low (# units)	Medium (#)	High (#)	Total (#)	Low (% units)	Medium (%)	High (%)
Eastern Cape	1	2	3	6	16.7	33.3	50.0
Free State	0	3	0	3	0.0	100.0	0.0
Gauteng	3	9	4	16	18.8	56.3	25.0
Kwa Zulu Natal	3	8	6	17	17.6	47.1	35.3
Limpopo	6	4	7	17	35.3	23.5	41.2
Mpumalanga	7	4	1	12	58.3	33.3	8.3
North West	1	11	2	14	7.1	78.6	14.3
Western Cape	3	6	1	10	30.0	60.0	10.0
Total	24	47	24	95	25.3	49.5	25.3
Excluded				6			

Those units with a weighted sum of risk scores below the IQR were assigned to the “low” risk group. Those units with a weighted sum of risk scores within the IQR were assigned to the “medium” risk group. Those units with a weighted sum of risk scores above the IQR were assigned to the “high” risk group

One ASF-affected unit of one compartment fell into the “high” risk group, while the other two ASF-affected units of the second compartment fell into in the “medium” risk group.

The units categorized as “high” risk based on the weighted sum of scores match the units categorized as “high” risk based on the unweighted sum of scores. Consistent with the results of the expert elicitation, the risk factors rated most frequently “high” among high-risk units were in the ‘human behavior & activities’ and ‘domestic pigs’ risk categories. “Insufficient boot and clothing biosecurity by animal health personnel” was rated “high” by all farm managers of the twenty-four (24) “high” risk units. Risk factors that were rated “high” by most of the “high” risk units in the ‘human behaviors & activities’ category were “insufficient boot and clothing biosecurity by external people,” “underreporting of suspect ASF cases,” and “improper hunting/ culling of wild suids inside the compartment.” Risk factors that were rated “high” by most of the “high” risk units in the ‘domestic pigs’ category were “un-tested introductions into the herd,” “entry of free-roaming pigs,” and “contact with free-roaming pigs.” Competent vector category risk factors were least frequently rated “high” among all units as well as among the “high” risk units.

**Fig. 3:**
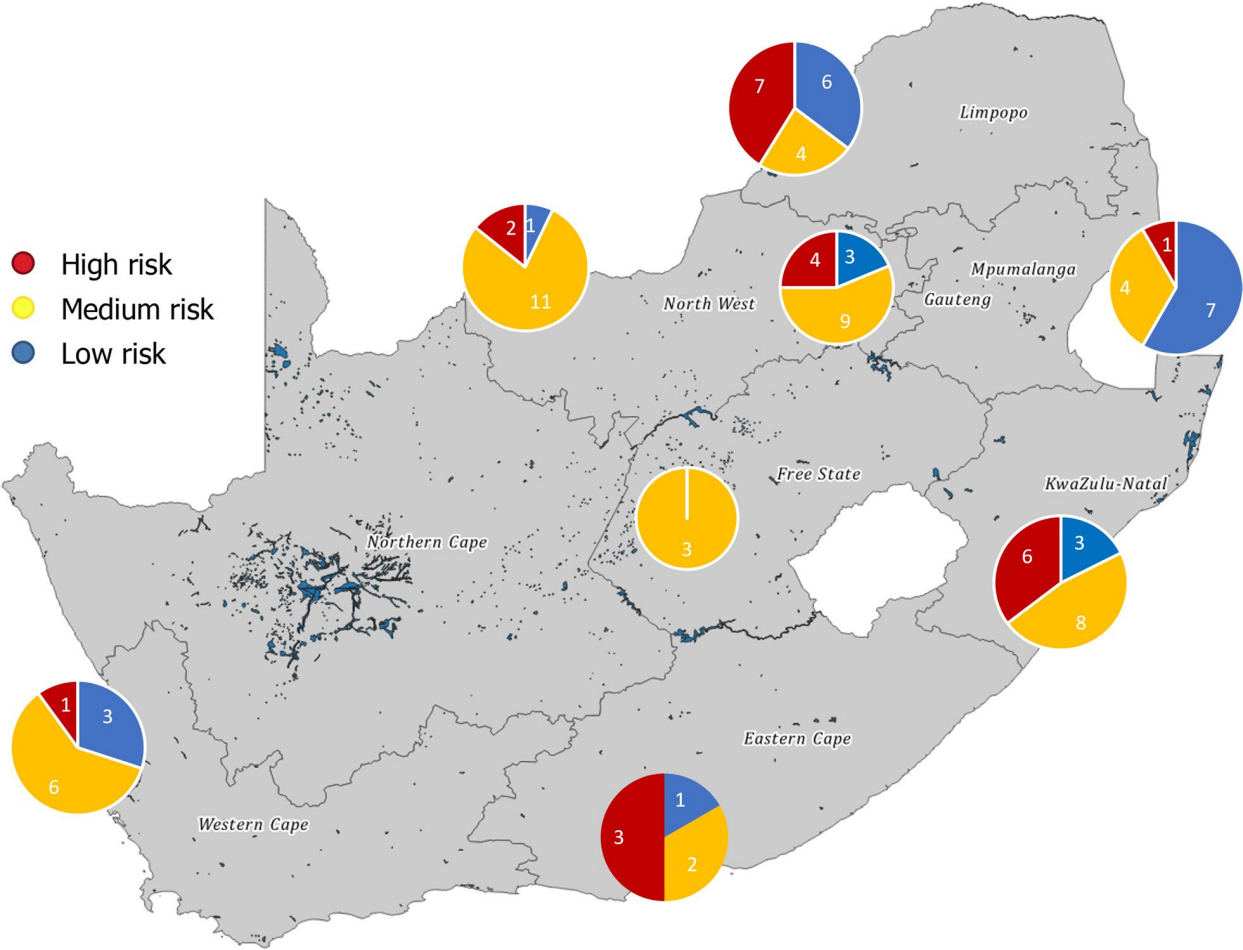
Compartment units categorized by risk at province level. High-risk units are clustered around the Gauteng–Limpopo border, with smaller clusters around the Kwa Zulu-Natal and Mpumalanga border, and in the southern part of the Eastern Cape. Limpopo has the highest number of high-risk units, while the Eastern Cape has the highest proportion of high-risk units

Limpopo had the highest number of “high” risk units (7 of 24) while the Eastern Cape had the highest proportion of “high” risk units among all of the units (3 of 6) (Fig. 3). There is an apparent clustering of “high” risk units around the border of Gauteng and Limpopo, around the border Kwa Zulu-Natal and Mpumalanga, and in the southern part of the Eastern Cape. Mpumalanga had the highest number of “low” risk units (7 of 24) and the highest proportion of “low” risk units (7 of 12).

### Principal component analysis and hierarchical clustering on principal component analysis

The Principal Component Analysis was performed on ninety-five (95) units, described by thirty-four (34) variables that align with the thirty-four (34) risk factors (Additional file 1). The actual eigenvalues and the proportion of variation explained by each eigenvalue is shown in Table 4 for the first two components. About 85.2% of the variation in the dataset is explained by the first two principal components (PCs) with the large majority of variance explained by the 1st PC (81.3%). The second component is responsible for the 3.9% of the variance. All other PCs have Eigenvalues inferior to 1 and therefore were not retained in the analysis [21].

**Table 4:** Eigen values of first 2 components

Principal components (PCs)	Eigenvalue	Percentage of variance	Cumulative percentage of variance
comp 1	27.65	81.32	81.32
comp 2	1.33	3.91	85.23

**Fig. 4:**
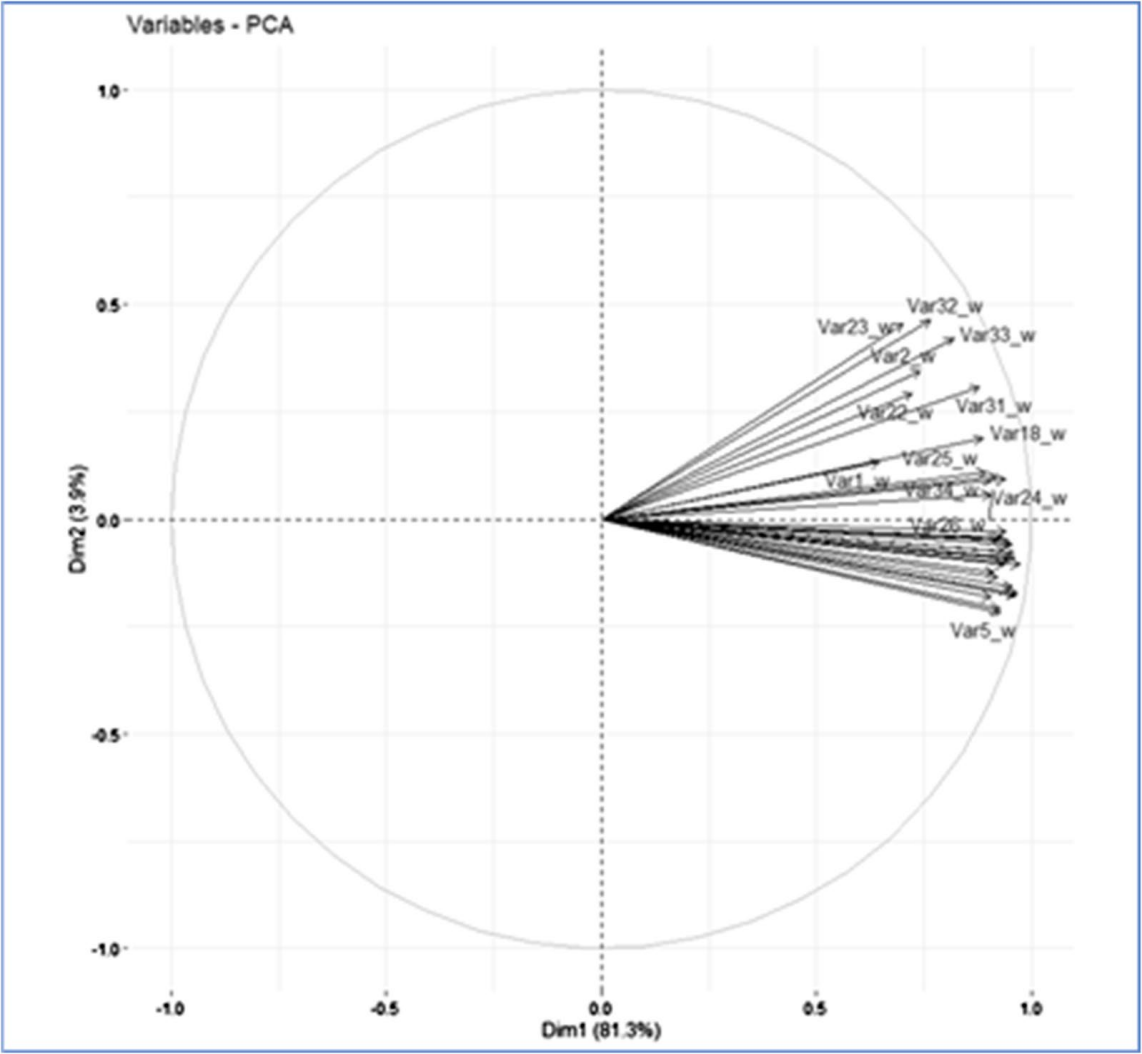
Correlation circle of active variables. The plot shows the correlation between a variable and the PCs

Figure 4 shows the correlation circle (correlation between a variable and a PC) of active variables. All variables are clustered together. All thirty-four (34) variables are strongly positively correlated with PC1 (correlation coefficient range 0.64–0.97) with all variables, with the exception of variables 1, 2, 18, 22–23, 25, 31–33 having a correlation coefficient larger than 0.90.

Eight variables are correlated with PC 2 of which 2 negatively correlated (Variables 5, 6; both with correlation coefficient of -0.21) and 6 positively correlated (variables 2, 22, 23, 31, 32, 33; range 0.29–0.46).

All variables contribute rather uniformly to PC 1 (Min: 1.51%, Max: 3.42%) while for PC 2 the extent of contribution varies considerably (Min: 0.06%, Max: 16.1%). The variables contributing more to PC 2 are Variables 2 (8.9%) (belonging to ‘domestic pigs’ category), 22 (6.4%), 23 (15.5%) (belonging to ‘competent vectors’ category), 31 (7.09%), 32 (16.1%), 33 (13.3%) (belonging to ‘fomites’ category). These variables (except for variable 22) match the variables identified by both farm managers and veterinarians as more relevant (i.e., rated as “high” or “medium” shown in Additional file 2).

**Fig. 5:**
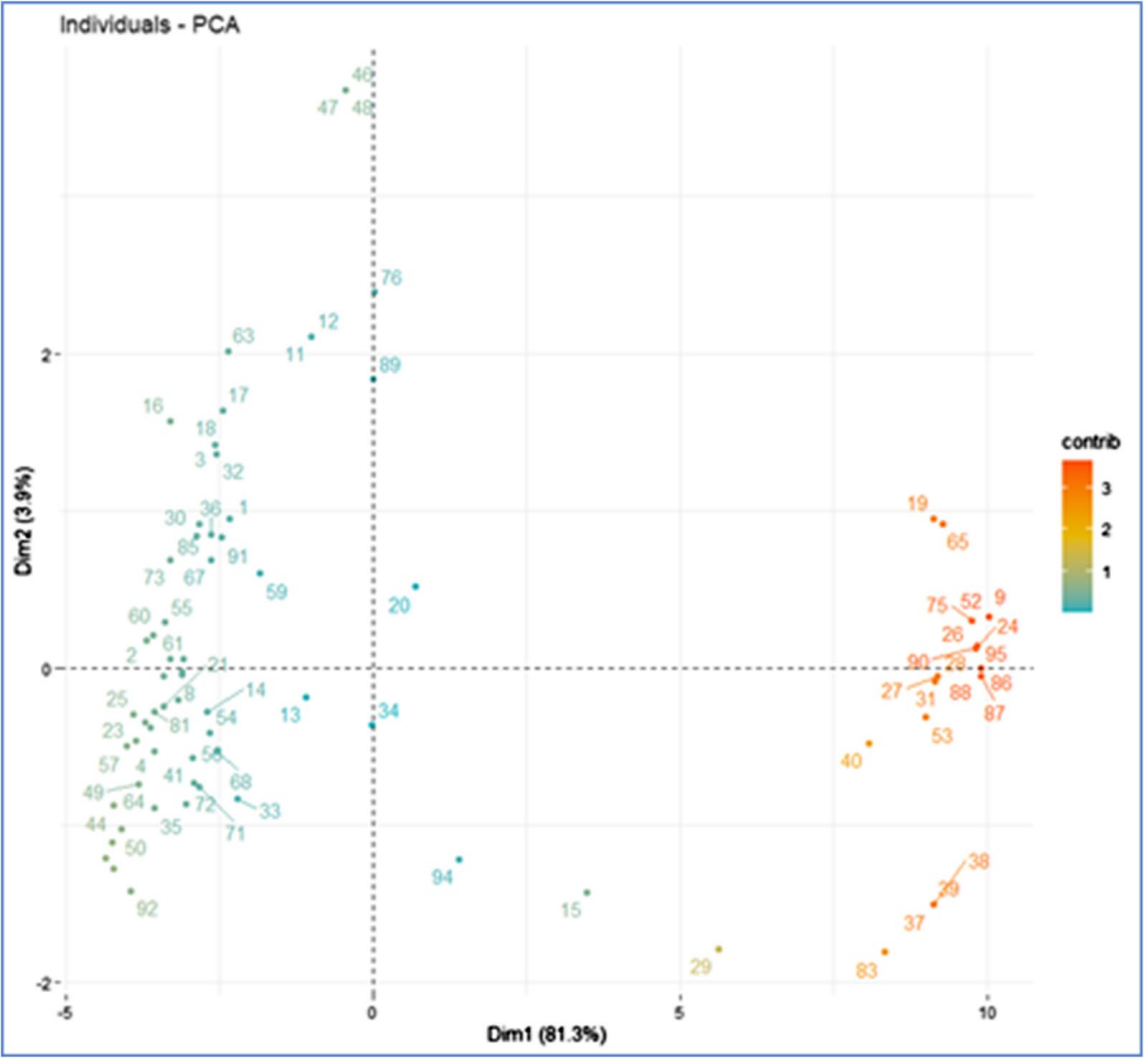
Plot of individual units in South Africa and their correlation with the 2 PCs. In this plot individuals that are similar are grouped together. Compartment units on the right section of the figure are the units contributing more to both PCs

Figure 5 shows the plot of individual units and their correlation with the 2 PCs. In this plot, individual units that are similar are grouped together. Compartment units on the right section of the Fig. 5 are the units contributing more to both PCs. The biplot graph in Additional file 4 confirms that the compartment units on the right section of both graphs are the units that take higher values of variables.

**Fig. 6:**
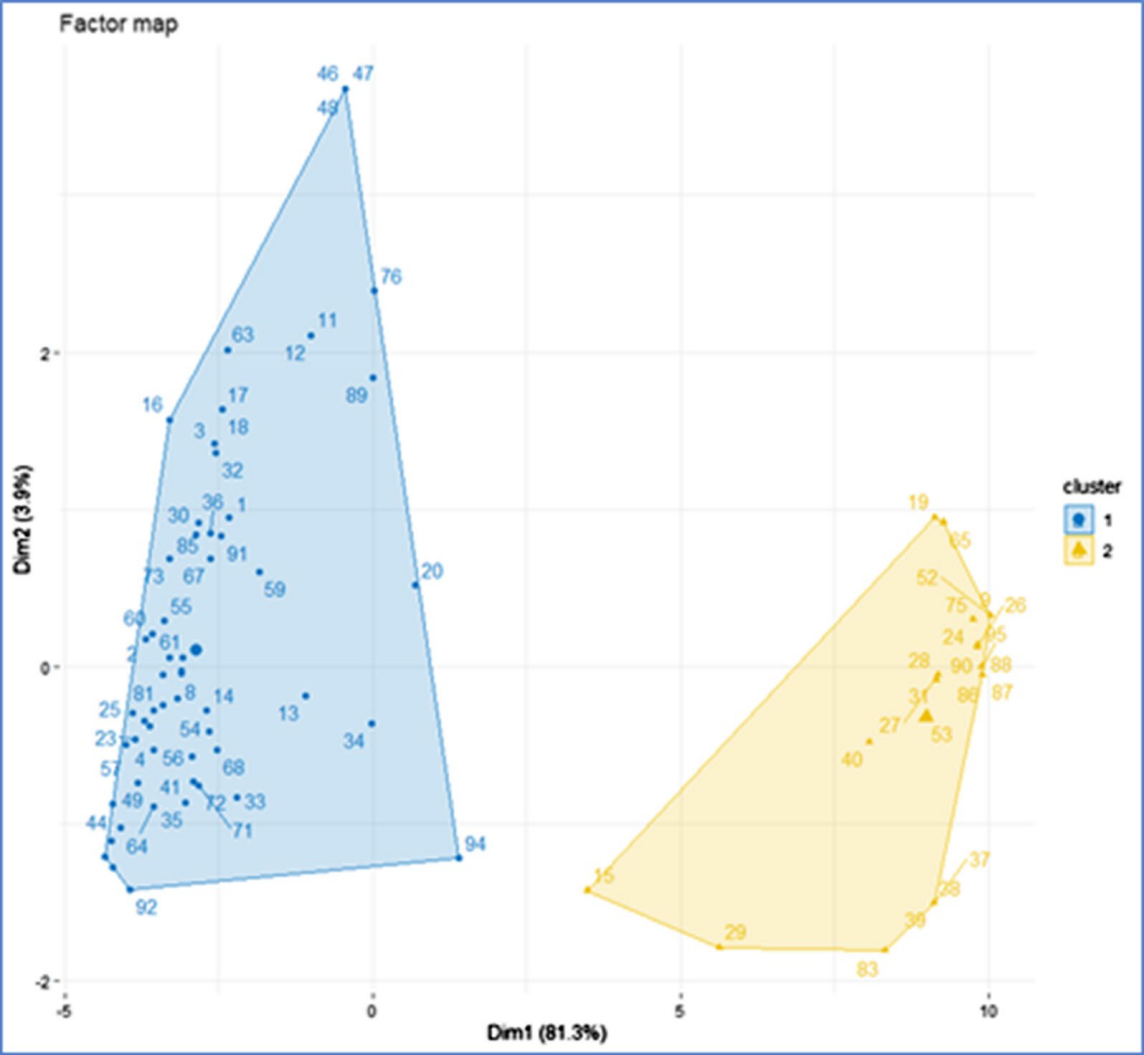
The hierarchical clustering on principal components analysis identified two main clusters of units on the principal components. Cluster 1 represents the low-medium risk cluster while Cluster 2 represents the high risk cluster

The Hierarchical Clustering on Principal Components analysis (Fig. 6) identified two main clusters of units on the principal components. Cluster 2 is made of all the compartment units with the highest risk scores (evaluated as “high” risk as per Table 3) while Cluster 1 is made of all units which were categorized as either “low” or “medium” risk with the exception of unit 94 which was categorized as “high” risk (and positioned in the low centre area of Fig. 6).

Cluster 2 (named “high” risk cluster) had a higher mean score for all the risk factor variables compared to cluster 1 (“low-medium” risk cluster). The variables contributing more to the cluster 2 (meaning that the specific variable mean score is higher than the mean of all variables in the same cluster) are variables 10–19 (all risk factors belonging to the ‘human behaviors and activities’ category). The variables contributing more to the cluster 1 are variables 1, 2 (both belonging to the ‘domestic pigs’ category), 10–19 (‘human behaviors and activities’ category) and 32 (‘fomites’ category).

## Discussion

This study investigates the drivers for ASF introduction into South Africa’s compartments and it categorizes them by risk of ASF introduction. Our results identify gaps not only in the compartment standards but also gaps in the knowledge of compartment personnel on ASF, its control, and prevention measures, where risk factors related to human behaviors & activities and domestic pigs are most critical. “Insufficient boot and clothing biosecurity by animal health personnel” is especially perceived as being relevant for the “high” risk units by the farm managers. While biosecurity standards can be improved and contingency plans can be prepared, they are of limited value if they are not understood or not followed by the target audience.

### Drivers of ASF introduction

The results of both the questionnaire, the expert elicitation, and the PCA confirm that drivers of disease related to the categories ‘human behaviors and activities’ and ‘domestic pigs’ present the highest risk to ASF virus introduction into compartment units in South Africa. The roles of wild pigs and competent vectors are considered relatively minimal.

These findings are consistent with those of other related studies. Fasina et al. [11] found that middlemen, traders, transporters, pig keepers who visit each other, and livestock field officers were identified as the human-related drivers of concern among rural farmers in Tanzania. Similarly, Huang et al. [17] found that pig density and human density were positively associated with regional ASF occurrence in West Africa. Pig movement and trade activity were identified as important factors for ASF spread, especially in West Africa [37]. Although there are differences in the swine industries and sylvatic cycles between the regions, the emphasis on the human behavioral component to the spread of ASF is shared. O’Hara et al. [29] found that certain high-risk behaviors by farm personnel on swine farms in Macedonia were correlated with high-risk biosecurity risk scores. Although the regional context in Macedonia differs from South Africa, the emphasis on commercial farm personnel behavior is consistent with the results of our study.

The proximity of most compartments to the ASF control zone is problematic. Most ASF-affected farms are also near the ASF control zone. It is therefore not surprising that “high” risk units were clustered near the ASF control zone. At the same time, with ASF continuing to spread in South Africa among non-compartment farms, it is also not surprising that proximity to farms with low biosecurity was frequently rated “high” or “medium” risk. Farms with low biosecurity present a risk for becoming ASF-infected, especially through human behaviors and activities. Therefore, farms with low biosecurity located in proximity to the compartment increase the risk for the units. This reported perception confirms the importance of strengthening biosecurity measures of units to reduce the risk presented by farms with poor biosecurity that are in proximity.

Since nearly all units have indoor only housing, this should limit the direct risk related to specific breaches in external biosecurity (i.e., external fences). However, the high ratings of certain risk factors related to human behavior and domestic pigs, such as insufficient decontamination of non-swine delivery vehicles, insufficient control of scavenger animals and pests, and high on-farm pig density, indicate that indoor housing alone is not considered sufficiently protective by respondents, especially when there are farms with low biosecurity or ASF-affected farms in proximity to the unit.

The control of scavenger animals both inside and outside the compartment premises is indicated as a top priority according to respondents. Indoor-only housing must be accompanied by robust control of scavengers and pests and robust vehicle and visitor management. Furthermore, high on-farm pig density is a risk factor for ASF even for indoor only pigs because more pigs inherently imply more cleaning & disinfection activities, more monitoring for clinical signs, and more likelihood of contact with ASF virus. These increases in activities imply higher likelihood of breaches in some biosecurity measures.

While there are reasonable explanations for the notable differences in the responses between the farm managers and the veterinarians, the differences also highlight a need for the two groups to be more aligned. It is possible that the veterinarians are not as aware as the farm managers of what additional standards are applied in the compartment. It is also possible that the questions were difficult to interpret. There may also be a discrepancy in what the farm managers and the veterinarians consider a contingency plan. Several veterinarians indicated that contingency plans must be developed. Inconsistencies between ratings of “high” or “medium” risk factors between farm managers and veterinarians highlight that perhaps the two groups are not aligned on the relevance of certain drivers of ASF virus introduction into compartments. The inconsistencies therefore warrant further reflection and engagement between the two groups to identify potential biases and to better understand and prioritize the more relevant drivers for ASF virus introduction into compartments.

### Categorization of units by risk

Notably, the perception of the relevance of risk factors to ASF introduction by the farm managers matches the expectations of the experts. This result is also in line with the results of the PCA and of the HCPC. The “high” risk units identified by “high” risk scores and the high-risk cluster from the HCPC match perfectly. The variables contributing more to the “high” risk cluster are those risk factors belonging to the ‘human behaviors and activities’ category. Therefore, attention should be paid to assessing and addressing risk factors related to ‘human behavior & activities’ and ‘domestic pigs’ in all units, but especially those that are identified as “high” risk. This is consistent with the understanding that ASF is increasingly an anthropogenic rather than a purely animal-health problem [32].

### Biosecurity standards and education

Even though the government standards and Pork360 standards were applied in most units, there is an apparent need for revision of the standards and thorough training of compartment personnel on the standards. The major gaps identified were the absence of a monitoring programme to assess the implementation of biosecurity measures, the appropriateness of the number of pigs tested and frequency of testing as well as the frequency and thoroughness of the audits. A closer look at the additional biosecurity measures in place in some units may inform improvements of the current standards, since those compartments that apply additional biosecurity standards may be more protected against ASF introduction than those that do not. While only three units were affected by ASF, a thorough field assessment of the affected units could help identify and address similar gaps in other units.

There is reported high compliance with the governmental standards for serological testing. However, for most units, no serological testing beyond the required testing is done. The reported low capacity of surveillance systems in some units to detect an occurrence of ASF early could be due to insufficient capacity of compartment personnel to recognize ASF clinical signs, lack of reporting of suspect cases to farm managers, and/ or insufficient testing. It is recommended that the sensitivity of the sampling and testing regime to detect positive cases be evaluated ad hoc to define cost-effective strategies.

There is also a lack of a clear contingency plan specific to ASF for most units. A clear contingency plan that is developed together between farm managers and veterinarians with dedicated resources and facilities could enhance compliance with the plan.

Furthermore, there is an apparent discrepancy between the compartment system in South Africa and the recommendations for a compartment system by the WOAH. The WOAH recommends that the entire pig value chain be included in a compartment, from feed production to consumer markets [33]. In contrast, South Africa’s compartment system is composed of individual swine enterprises and four artificial insemination stations and is therefore missing important components such as feed in the overall compartment concept.

While a revision of the standards is considered, the capacity of farm managers and compartment personnel to apply trainings on ASF and biosecurity can also be improved, considering also that human behaviors and activities are found to be the most relevant to ASF virus introduction into units. Since the number of personnel in contact with pigs daily is greater than seven for over 75% of units, the training and knowledge of these personnel on ASF is a critical aspect of biosecurity to consider. It is possible that the more personnel that are in contact with pigs, while necessary for efficiency of operations, the higher the risk of reduced or non-compliance with biosecurity measures and thus ASF introduction. Based on the results of this study, the education and awareness-raising efforts should include not only compartment personnel but also owners and personnel of the non-compartment farms that are in proximity to compartments to ensure that the risk of disease spread between the two farming systems is addressed.

The degree in uncertainty among the farm managers in their answers to the questions in this section also demonstrated potential gaps in their knowledge on ASF modes of transmission, clinical signs, prevention and control strategies and therefore potential gaps in their capacity to assess compartment personnel for the same capacities. These gaps in capacity were reflected in the gaps in the likelihood of reporting of suspect ASF cases, which is critical to early detection and control of the disease.

### Limitations

The questionnaire results completed for multiple units were replicated so that each data point represented a single unit. The denominator of all the analyses is therefore the units and not the respondents. While criteria for grouping compartments in a single questionnaire were clearly outlined for the veterinarians, these were not outlined for farm managers, since each unit is assumed to have a unique farm manager. Nevertheless, 12% of farm managers filled out a single questionnaire for multiple compartment units.

The approach to use the units as the denominator has some limitations as well, since it is assumed that respondents intended to submit the same results for each unit of the units that were grouped together in a single questionnaire. Since the perceptions of only thirteen (13) veterinarians are considered for a larger number of units, individual bias may be reflected in the results. We can assume the responses of the farm managers are relatively specific while recognizing the limitation of the approach to use the unit as the denominator for the analyses.

Despite differences in responses between the farm managers and veterinarians to some of the questions, the approaches used in this study are robust considering that the stakeholders most appropriate to evaluate the relevance of risk factors were targeted. Targeting both farm managers and veterinarians is a strength to the approach where inconsistencies can be highlighted and used by SAPPO to align the two groups more closely.

Further limitations of the study include that it is perception-based, relying on the compliance and honesty of the respondents. The remote nature of the study prohibited in-person investigation of the compartments and in-person interviews, which may otherwise have provided more objective and detailed insights. There was limited opportunity for respondents to expand on the details of their responses that would otherwise arise in an interview. Nevertheless, the data collected provides us with a consistent understanding of the situation for the compartment system.

While interpretations of the results for all compartment units are summarized here, it is important to note that critical units should be evaluated individually. The combination of “high” risk factors can have an impact on the risk of ASF introduction that may be greater than the impact of a single “high” risk factor.

## Conclusions

With ASF being a great threat to swine industries globally, industries are looking toward implementing prevention measures like compartmentalization to allow for business continuity in the face of an ASF-introduction. ASF compartments also increase the overall biosecurity of the industry. Given the limited number of existing compartment systems for swine, the organization of the compartment system in South Africa can be considered an effective approach to protecting the pig value chain from transboundary animal diseases like ASF once they have entered a country. The ASF breaches in the three units provide an opportunity to learn how to identify and address the gaps in a compartment system, as shown by this study. In addition, particular attention should be given to the interactions between the formal pig compartment system and non-compartment farms which could represent a risk for disease introduction in the event of breaches in biosecurity. Strengthening of the compartment system in South Africa based on this study can act as a model for swine industries globally to pursue compartmentalization and thus safeguard their businesses in the face of ASF.

Recommendations for improved biosecurity are not only important for those units identified as higher risk but also equally important for all units. Logically, higher risk units can be identified and assessed in closer detail.

The main recommendation is for the governmental compartment standards to be reviewed and updated if necessary for the following elements:

- The sensitivity of the ASF sampling and testing strategy, especially for suspect ASF-infected units
- The frequency and thoroughness of audits
  - Every compartment should have an internal auditing mechanism is place in addition to the required inspections for compartments and members of the Pork360 accreditation scheme.
- A monitoring programme to assess the implementation of biosecurity measures according to SOPs as a requirement.
- An ASF contingency plan as a requirement.
  - Due to the potential consequences of not having an ASF contingency plan in place, it is worth while to invest resources to develop a plan that can be adapted to each compartment unit. The contingency plan should be specific to ASF with dedicated finances prior to an outbreak.

Furthermore, more compartments should be encouraged to join the Pork360 accreditation scheme. A revision of the compartment system should be considered for consistency with WOAH guidelines [30, 33]. Through alignment with the WOAH guidelines, the compartment system may be re-structured. Rather than many compartments linked to few slaughterhouses, a more biosecure network would have fewer compartments with biosecure connections throughout the pig value chain. By revising the criteria for compartment registration to be more rigorous, fewer compartments would be approved going forward.

Biosecurity measures that can be strengthened to reduce the risk of ASF introduction are listed in Additional file 5 by risk factor for the top risk factors consistently rated “high” or “medium” between the two groups.

Knowledge of farm managers and compartment personnel on pig diseases especially ASF is crucial for the strengthening of biosecurity measures of units. To enhance compliance with biosecurity measures and thus control the disease, close engagement with all stakeholders linked to the compartments is needed, including but not limited to animal health personnel, farm managers, and compartment personnel [32]. Capacity development approaches to training of compartment personnel on ASF and biosecurity can form the foundation for the internalization of knowledge at the individual and the organizational level.

## Methods

The approach to the study included four sequential steps. First, an online questionnaire was developed and shared with compartment veterinarians and farm managers to investigate their perception with regard to the relevance of risk factors for ASF introduction for each unit. Afterwards, an expert elicitation with local and international experts was conducted to assign weights to the categories of risk factors. This was done with the view of contextualizing the risk factors according to the local ASF situation, and thus producing more accurate variables. The weighted risk factors were used to categorize units into risk-levels according to the IQR. These categorizations were confirmed though a principal component analysis (PCA) and hierarchical clustering on the principal component analysis.

### Identification of ASF risk factors and online questionnaire

An online questionnaire of the compartment veterinarians and farm managers was used to investigate their perception with regard to the relevance of risk factors for ASF introduction for each compartment unit. Ninety-seven (97) farm managers and thirteen (13) consulting veterinarians of all one hundred twenty-seven (127) compartment units were solicited to complete the questionnaire between May and June 2021. The development of the online questionnaire for this study occurred in three stages.

First, a preliminary questionnaire was developed for completion by SAPPO in order to gain an overall understanding of the compartment characteristics and of the ASF presence in compartment area. Information on organization of the compartments, geographical features and ASF presence (confirmed by veterinary authority), stakeholders and management systems, and available compartment documents/protocols was gathered.

A risk factor matrix was then developed including potentially relevant risk factors for ASF introduction in the compartments in the South African context and the recommended preventive measure for each risk factor by category (Additional file 6). The identification of risk factors and preventive measures was based on a review of selected manuscripts and biosecurity guidelines and reports [2, 5, 9, 11, 14, 34]. The matrix and list of risk factors were reviewed and validated by SAPPO.

Finally, two online questionnaires were developed using SurveyMonkey and pilot tested with similar target subjects. The questionnaires were revised based on the results of the pilot tests and then distributed to the farm managers and the veterinarians respectively. The questionnaire for the farm managers was designed for one compartment per questionnaire, while the questionnaire for the veterinarians was designed for multiple compartments per questionnaire based on a described set of criteria. This was necessary because veterinarians are responsible for up to twenty-five units that are potentially under different ownership. Data on demographics, farm characteristics, management and biosecurity, sanitary situation, knowledge of ASF, internal surveillance system for ASF, and perception of relevance of risk factors to ASF introduction were collected. While the two questionnaires were for the most part similar in order to compare the answers between the two respondent types, some questions were asked only to one respondent group based on their expected knowledge areas to gain further insights.

For the assessment of the relevance of drivers of disease introduction the following categorical scales of evaluation were used:

- **Negligible = **risk factor is most likely absent or insignificant; therefore, the influence of this risk factor towards the risk of ASF introduction is perceived to be negligible.
- **Low = **risk factor presence is a rare probability (but cannot be excluded); the influence of this risk factor towards the risk of introduction is perceived to be low;
- **Medium = **risk factor presence is a concrete probability; the influence of this risk factor towards the risk of introduction is perceived to be medium;
- **High = **risk factor presence is a highly probable; the influence of this risk factor towards the risk of introduction is perceived to be high;
- **NA (not applicable) = **you don’t feel comfortable providing an answer for this risk factor

“Non-negligible” as used in this report therefore means that the probability of a specific risk factor presence cannot be, at a minimum, excluded. Uncertainty in questionnaire responses was estimated by asking the respondents to rate their confidence in their answers for certain sections of the questionnaire. Answer choices included “not at all confident”, “confident”, and “very confident”.

A descriptive analysis was conducted separately on the data from farm managers and the data from the veterinarians using IBM® SPSS® Statistics 26 [19]. Questionnaires submitted on grouped compartment units were replicated for each unit covered in a single questionnaire, so that the denominator value was the number of compartment units (101 for farm managers, 118 for veterinarians) when relevant for the analyses.

The median of responses to each question was calculated as the measure of central tendency rather than the mean because of the data skewedness. The categorical ratings were assigned a number in order to make the calculations using the following scales where relevant:

- Basic = 1, medium = 2, good = 3
- Negligible = 1, low = 2, medium = 3, high = 4

To rank the top risk factors among farm managers and veterinarians, frequencies and percentages of individual ratings for each risk factor were calculated using Microsoft Excel. Risk factors were ranked from highest to lowest risk based on the percentage of units that rated the risk factor “high” or “medium” separately by farm managers and veterinarians. The rankings between farm managers and veterinarians were compared for consistency.

### Expert elicitation and categorization of compartment units by risk

An expert elicitation was conducted and based on a Delphi approach [28] to gather the opinion of professionals with recognized scientific expertise or experience in ASF epidemiology to weigh categories of risk factors involved in ASF introduction in compartment units in South Africa. Eleven international experts mostly working in South Africa and with relevant scientific backgrounds were asked in two rounds between November 2021 and January 2022 to weigh five categories of risk factors as defined for the online questionnaire considering the South African context with a rating of uncertainty from 1 to 3 (Additional file 7). The template shared with experts for weighing of risk factor categories can be found in Additional file 8.

Experts were provided with background information on compartment organization and management, including biosecurity and risk management practices. Experts were asked to weigh each category of risk factors using the Las Vegas technique [15], they were asked to distribute one hundred (100) points between the five categories of risk factors according to the importance of each group to ASF introduction into a compartment unit in South Africa. The risk factors within the categories and definitions of uncertainty scores were provided in the Excel file for reference.

The anonymized first round of results and a summary of the comments were shared with the experts to inform their second round elicitations according to Delphi approach methods [8, 28]. The median weight of the second round elicitation was taken for the final weight of the category of risk factors.

Uncertainty scores were used to calculate a weighted average for each category. A consensus on the final median weights was reached by sharing with the experts for final agreement.

To assign a weighted risk score to each compartment unit, the weighted score of each category of risk factors was multiplied by the sum of the ratings for that category of risk factors as determined by the respondent for that unit.

The following formula was used:

$${\text{WSR}}_{{\text{a}}} = \frac{1}{100}*\mathop \sum \limits_{e = 1}^{11} {\text{S}}_{{\text{a}}} C_{{\text{e}}} *WC_{{\text{e}}}$$

with WSR_a_ being the weighted score for each risk factor, S_a_C_e_ being the score given by the experts for the category e and risk factor a, and WC_e_, being the relative weight of the category e.

This weighting system was only applied to the farm managers results. Each farm manager is unique to the compartment unit and is assumed to be engaged in unit-specific operations more regularly. They are expected to be familiar with and responsible for adherence to the SOPs and to have oversight over compartment personnel responsibilities.

The higher the weighted risk score, the higher the influence of drivers of disease introduction towards the compartment unit, as perceived by the farms managers. Since the weighted risk scores had a highly skewed distribution, the IQR of the weighted risk scores among all compartment units was used to assign compartment units into risk categories. The IQR is the spread difference between the 75th and 25th percentiles of the data. The risk categories were defined as follows:

- High risk group—Weighted risk score above the IQR
- Medium risk group—Weighted risk score within the IQR
- Low risk group—Weighted risk score below the IQR

Units where farm managers rated ten or more risk factors as NA were excluded from the analysis. The results of these groupings of compartment units into risk categories were compared with the results of the principal component analysis for consistency (see “Principal component analysis and hierarchical clustering on principal component analysis” section).

Compartment units by risk category were mapped using latitude and longitude coordinates reported by farmer managers and by province using QGIS 3.22.3 [35]. Administrative units were downloaded from DIVA-GIS [16].

### Principal component analysis and hierarchical clustering on principal component analysis

Principal component analysis (PCA) was used to categorize compartment units into risk levels. The “active” variables contributing to the calculations in the PCA were the drivers of ASF introduction only. PCA allows to summarize and visualize the information contained in a multivariate dataset and to express the information as a set of new variables called principal components which correspond to a linear combination of the original variables. The visualization of the PCA results allows to interpret the association between the selected active variables and their contributions to the identified components [21]. Key outputs from the PCA are, among others, the number of components explaining the total variance in the dataset and the Eigenvalues expressing the amount of variation retained by each principal component. The correlation between a specific variable and each component is visualized through a correlation circle and the biplot graph where both variables and compartment units are fictitiously superimposed [18].

We then applied hierarchical clustering on principal components (HCPC) method to identify group of similar compartment units according to similar patterns of variables responses (drivers of ASF introduction) with the dataset of reference, e.g. compartment units with similar profiles (answers related to the drivers of ASF introduction) would be clustered together [22]. The main output would be the identification of clusters of compartment units on the principal components visualized through a factor map. PCA and HCPC were performed in R Studio [37] using the FactoMineR [24] and factoextra [23] packages.

## Supplementary Information

**Additional file 1.** Risk factors relevant to ASF introduction into compartments in South Africaby category.

**Additional file 2.** Top rated risk factors.

**Additional file 3.** Perception of relevance of risk factors.

**Additional file 4.** Biplot.

**Additional file 5.** Recommendation for improved biosecurity in compartment units.

**Additional file 6.** Risk factor matrix.

**Additional file 7.** Rating of Uncertainty Scores in Expert Elicitation.

**Additional file 8.** Template for weighing of risk factor categories for the expert elicitation.

## Data Availability

The datasets generated and analysed during the current study are not publicly available due to confidentiality of the compartment units but are available from the corresponding author on reasonable request.

## Abbreviations

ASF: African Swine Fever
IQR: Interquartile range
PC: Principal Component
PCA: Principal Component Analysis
SAPPO: South African Pork Producers' Organisation
SOP: Standard operating procedure
VPN: Veterinary Procedural Notice
WOAH: World Organisation for Animal Health
