# Supplementary material for: Analysis of the drivers of ASF introduction into the officially approved pig compartments in South Africa and implications for the revision of biosecurity standards
Source: Porcine Health Manag. 2022 Oct 6;8:43. doi: 10.1186/s40813-022-00286-7 (PMC9540751; doi:10.1186/s40813-022-00286-7)
Supplement: Supplementary file 1 — Additional file 1. Risk factors relevant to ASF introduction into compartments in South Africaby category. [file 40813_2022_286_MOESM1_ESM.pdf]

Additional File 1. Risk factors relevant to ASF introduction into compartments in South Africa by category

Category	No.	Risk factor	Proxy
Domestic pigs	1	High on-farm domestic pig density	On-farm pig density
	2	Farms with poor biosecurity (e.g. backyard farm) located outside but in proximity to the compartment (< 5km)	Proximity to farms with poor biosecurity
	3	Proximity of the compartment (< 5 km) to a farm that was infected with ASF within the last three months	Proximity to ASF-affected farms
	4	Introduction of new pigs to the compartment without testing for ASF virus or quarantine	Un-tested introductions into the herd
	5	Use of boars from outside the compartment for breeding that are not tested for ASF virus	Use of un-tested breeding boars
	6	Use of pig semen, ova or embryos from a non-ASF-free certified source(s)	Use of uncertified genetic material
	7	Entry of free-roaming pigs into the compartment premises due to improper fencing	Entry of free-roaming pigs
	8	Direct and indirect contact between pigs within the compartment premises and free-roaming pigs outside the compartment premises due to improper fencing	Contact with free-roaming pigs
	9	Return of live pigs to the compartment following presentation to markets or auctions	Return of live pigs
Human behaviors and activities	10	Insufficient boot cleaning & disinfection or boot & clothes changing of external people (e.g. pig traders, middlemen, transporters) upon entering the pig sites in the compartment	Insufficient boot and clothing biosecurity by external people
	11	Insufficient boot cleaning & disinfection or boot & clothes changing of livestock field officers/ veterinarians/ para-veterinarians upon entering the pig sites in the compartment	Insufficient boot and clothing biosecurity by animal health personnel
	12	Insufficient cleaning & disinfection or changing of clothes & boots, facilities and equipment of compartment personnel that are in contact with pigs	Insufficient cleaning & disinfection of boots, clothes, facilities, and equipment
	13	Feeding of non-cooked or under cooked swill i.e. commercial or household food waste within the compartment	Feeding of food waste
	14	Underreporting of suspected ASF cases due to various factors e.g. insufficient capacity to recognize ASF clinical signs, lack of an easy reporting system	Underreporting of suspect ASF cases

	15	Euthanasia/ culling of sick pigs within the premises of the compartment without proper disposal of carcasses and offal	Improper carcass disposal of sick pigs
	16	Slaughtering of pigs within the premises of the compartment for human consumption without veterinary supervision or without proper disposal of carcasses and offal	Improper on-site slaughter
	17	Hunting/ culling of wild suids within the compartment premises without proper containment and disposal of carcasses and offal resulting in presence of contaminated materials on the site	Improper hunting/ culling of wild suids inside the compartment
	18	Hunting/ culling of wild suids outside but in proximity to the compartment premises (< 5km) without proper disposal of carcasses and offal resulting in presence of contaminated materials on the site	Improper hunting/ culling of wild suids in proximity to the compartment
	19	Compartment personnel taking meals regularly outside areas specifically reserved for dining	Meals outside designated areas
Wild suids	20	Wild suid entry into the compartment premises due to improper fencing	Wild suid entry
	21	Direct and indirect contact between pigs within the compartment premises and wild suids outside the compartment premises due to improper fencing	Contact with wild suids
Competent vectors	22	Presence of the tick vector (i.e. tampans) in and around (< 5km) the compartment premises without tick control measures	Tick vectors
	23	Presence of biting flies (e.g. Stomoxys calcitrans) in and around (< 5km) the compartment premises without fly control measures	Biting flies
Fomites	24	Insufficient decontamination of swine transport vehicles entering & leaving the compartment premises	Insufficient decontamination of swine transport vehicles
	25	Insufficient decontamination of non-swine delivery vehicles (e.g. supplier of feed, equipment, health products) entering & leaving the compartment premises	Insufficient decontamination of non-swine delivery vehicles
	26	Insufficient decontamination of own tractors & lawnmowers entering the compartment premises	Insufficient decontamination of own tractors & lawnmowers
	27	Transport of pigs from multiple non-compartment sources in the same vehicle to compartment	Same-vehicle transport
	28	The same vehicle of the compartment used to transport pigs from the compartment to the abattoir is also used for internal movement of pigs, feed or raw	Abattoir transport

		materials without sufficient decontamination between shipments	
	29	Use of feed (e.g. fresh grass, seeds, crops) or bedding originating from areas in which wild suids have had access	Contaminated feed or bedding
	30	Improper disposal of domestic pig carcasses and manure within the compartment premises	Improper disposal of carcasses and manure
	31	Insufficient control of scavenger animals (e.g. sacred ibis, feral cats, feral dogs, feral pigs) within the compartment premises	Insufficient control of scavenger animals within the compartment
	32	Insufficient control of scavenger animals (e.g. sacred ibis, feral cats, feral dogs, feral pigs) outside but in proximity to (< 5km) the compartment premises	Insufficient control of scavenger animals in proximity to the compartment
	33	Insufficient pest (e.g. mice, rats, birds) control within the compartment premises	Insufficient pest control
	34	Regular presence of pets (e.g. dogs, cats) within the compartment premises	Regular presence of pets
