# Supplementary material for: Analysis of the drivers of ASF introduction into the officially approved pig compartments in South Africa and implications for the revision of biosecurity standards
Source: Porcine Health Manag. 2022 Oct 6;8:43. doi: 10.1186/s40813-022-00286-7 (PMC9540751; doi:10.1186/s40813-022-00286-7)
Supplement: Supplementary file 2 — Additional file 2. Top rated risk factors. [file 40813_2022_286_MOESM2_ESM.pdf]

Additional File 2 – Top rated risk factors

Top ten risk factors rated most-frequently as “high” or “medium” among farm managers and veterinarians. Eight of ten top risk factors are shared between the farm managers and the veterinarians, shown in bold.

Ranking # (high to low)	Risk Factor	% of units* rated high or medium farm managers	% of units** rated high or medium among veterinarians
1	**02**	42.6	34.7
2	**01**	28.7	42.3
3	**32**	37.6	28.8
4	**18**	27.8	15.3
5	**31**	28.7	12.7
6	**23**	29.7	11
7	**33**	29.7	11
8	12	24.8	14.4
9	**03**	25.8	12.7
10	25	26.7	10.2

*Percentage is shown out of 101 total responding compartment units.
**Percentage is shown out of 118 total responding compartment units.
