# Supplementary material for: Analysis of the drivers of ASF introduction into the officially approved pig compartments in South Africa and implications for the revision of biosecurity standards
Source: Porcine Health Manag. 2022 Oct 6;8:43. doi: 10.1186/s40813-022-00286-7 (PMC9540751; doi:10.1186/s40813-022-00286-7)
Supplement: Supplementary file 3 — Additional file 3. Perception of relevance of risk factors. [file 40813_2022_286_MOESM3_ESM.pdf]

Additional File 3 – Perception of relevance of risk factors

The percentages of compartment units by rating of perceived risk of risk factors reported by farm managers and veterinarians are shown in the table below. The sum of the % of high and medium score for each driver objectively identifies those factors that, according to the scale definitions, have a concrete possibility to be present or whose presence is highly probable in the compartment unit. The top ten risk factors reported by farm managers as “high” or “medium” and the top eleven risk factors reported by veterinarians are highlighted in orange in Table 3 to emphasize those risk factors that were rated as having a perceived higher risk.

In the “domestic pigs” category, risk factors #1, #2, and #3 were consistently in the top ranking for “high” or “medium” between the farm managers and the veterinarians. In the “human behaviors & activities” category, risk factor #18 was consistently in the top ranking for “high” or “medium” between the farm managers and the veterinarians. Wild suid risk factors were generally perceived as “negligible” among fam managers and veterinarians. Competent vector risk factors were frequently perceived as non-negligible among farm managers and veterinarians. Risk factor #23 was consistently in the top ranking for “high” or “medium” between the farm managers and the veterinarians and was frequently ranked as non-negligible among farm managers (59.4 % of units) and veterinarians (58.5 % of units). In the “fomite” category, risk factors #31, #32, and #33 were consistently in the top ranking for “high” or “medium” between the farm managers and the veterinarians. Risk factors #24 and #26 were more frequently rated “high” or “medium” by veterinarians whereas risk factors #25 was more frequently rated “high” or “medium” by farm managers.

Table. Percentage of compartment units by risk factor rating for ASF introduction into compartment units. Top risk factors shared by farm managers and veterinarians are shown in bold.

	Farm Managers*							Veterinarians**						
**Risk factor**	High	Med-ium	Low	Neg.	NA	High & Med-ium	Med-ian	High	Med-ium	Low	Neg.	NA	High & Med-ium	Med-ian
1.	20.8	7.9	29.7	37.6	4	**28.7**	2	23.7	18.6	16.9	40.7	0	**42.3**	2
2.	32.7	9.9	17.8	35.6	4	**42.6**	2	16.9	17.8	31.4	30.5	3.4	**34.7**	2
3.	22.8	3	5.9	64.4	4	**25.8**	1	6.8	5.9	5.9	66.1	15.3	**12.7**	1
4.	25.7	0	8.9	57.4	7.9	25.7	1	0.8	1.7	11	80.5	5.9	2.5	1
5.	21.8	0	1	64.4	12.9	21.8	1	0	1.7	0	85.6	12.7	1.7	1
6.	20.8	1	2	63.4	12.9	21.8	1	0	0.8	0	86.4	12.7	0.8	1
7.	23.8	0	0	67.3	8.9	23.8	1	3.4	5.9	25.4	65.3	0	9.3	1
8.	23.8	0	2	65.3	8.9	23.8	1	3.4	4.2	26.3	66.1	0	7.6	1
9.	21.8	1	1	65.3	10.9	22.8	1	0.8	0	0	86.4	12.7	0.8	1
10.	22.8	2	24.8	49.5	1	24.8	1.5	4.2	1.7	47.5	46.6	0	5.9	2
11.	23.8	0	18.8	57.4	0	23.8	1	0.8	4.2	33.9	61	0	5	1
12.	21.8	3	21.8	53.5	0	24.8	1	0	14.4	43.2	42.4	0	**14.4**	2
13.	20.8	1	4	65.3	8.9	21.8	1	2.5	0.8	5.9	80.5	10.2	3.3	1
14.	23.8	5	16.8	51.5	3	**28.8**	1	2.5	0.8	15.3	81.4	0	3.3	1

15.	18.8	3	11.9	60.4	5.9	21.8	1	0	2.5	14.4	77.1	5.9	2.5	1
16.	17.8	2	6.9	65.3	7.9	19.8	1	2.5	0	4.2	83.9	9.3	2.5	1
17.	22.8	0	3	65.3	8.9	22.8	1	3.4	0	12.7	78.8	5.1	3.4	1
18.	23.8	4	13.9	50.5	7.9	**27.8**	1	11.9	3.4	44.1	40.7	0	**15.3**	2
19.	21.8	2	15.8	57.4	3	23.8	1	0	5.9	45.8	44.1	4.2	5.9	2
20.	20.8	0	9.9	67.3	2	20.8	1	0	2.5	47.5	50	0	2.5	1.5
21.	20.8	1	4	72.3	2	21.8	1	0	2.5	30.5	66.9	0	2.5	1
22.	16.8	5.9	27.7	45.5	4	22.7	2	2.5	7.6	22.9	66.9	0	10.1	1
23.	11.9	17.8	29.7	38.6	2	**29.7**	2	4.2	6.8	47.5	36.4	5.1	**11**	2
24.	21.8	3	27.7	46.5	1	24.8	2	3.4	7.6	54.2	34.7	0	**11**	2
25.	19.8	6.9	32.7	38.6	2	**26.7**	2	0	10.2	56.8	33.1	0	10.2	2
26.	18.8	3	38.6	39.6	0	21.8	2	0	12.7	54.2	33.1	0	**12.7**	2
27.	20.8	3	6.9	66.3	3	23.8	1	0	0.8	9.3	77.1	12.7	0.8	1
28.	21.8	3	8.9	60.4	5.9	24.8	1	0	0.8	29.7	55.9	13.6	0.8	1
29.	20.8	1	56.4	13.9	7.9	21.8	1	0	0.8	15.3	78	5.9	0.8	1
30.	18.8	3	8.9	65.3	4	21.8	1	0	0	16.9	78.8	4.2	0.0	1

31.	25.7	3	24.8	44.6	2	**28.7**	2	3.4	9.3	43.2	41.5	2.5	**12.7**	2
32.	21.8	15.8	34.7	27.7	0	**37.6**	2	8.5	20.3	51.7	16.9	2.5	**28.8**	2
33.	17.8	11.9	35.6	33.7	1	**29.7**	2	4.2	6.8	57.6	31.4	0	**11**	2
34.	18.8	5.9	11.9	59.4	4	24.7	1	3.4	2.5	16.9	64.4	12.7	5.9	1

*Percentage is shown out of 101 total responding compartment units.

**Percentage is shown out of 118 total responding compartment units.
