# Supplementary figures and images for: Analysis of the drivers of ASF introduction into the officially approved pig compartments in South Africa and implications for the revision of biosecurity standards
Source: Porcine Health Manag. 2022 Oct 6;8:43. doi: 10.1186/s40813-022-00286-7 (PMC9540751; doi:10.1186/s40813-022-00286-7)

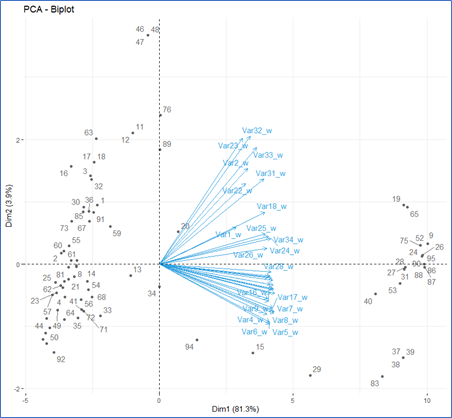

Supplement: Supplementary file 4 — Additional file 4. Biplot. [file 40813_2022_286_MOESM4_ESM.tif]
