# Supplementary material for: Analysis of the drivers of ASF introduction into the officially approved pig compartments in South Africa and implications for the revision of biosecurity standards
Source: Porcine Health Manag. 2022 Oct 6;8:43. doi: 10.1186/s40813-022-00286-7 (PMC9540751; doi:10.1186/s40813-022-00286-7)
Supplement: Supplementary file 5 — Additional file 5. Recommendation for improved biosecurity in compartment units. [file 40813_2022_286_MOESM5_ESM.pdf]

Additional File 5 - Recommendation for improved biosecurity in compartment units

Recommendations for improving biosecurity for top rated risk factors in order of average % units rated "high" or "medium" from highest to lowest among farm managers and veterinarians

Risk factor	Recommendation for improved biosecurity in compartment units
02.	• **Keep pigs indoors** at all times while maintaining an enriching environment to optimize animal welfare.<br>o For units that must allow outdoor access for pigs due to animal welfare requirements, outdoor access can be resumed once ASF has stopped spreading in domestic pigs in the region and farms have recovered from outbreaks. Active surveillance of animals with outdoor access for ASF will be essential in the recovery period.<br>• **Fence in compartment unit** with non-porous fencing.<br>o All pig production areas / units must be totally and effectively fenced (pig proof) with a fence that is a minimum of 1.8m high.<br>o All access gates must be closed and locked at all times.<br>o Limited access shall be allowed.<br>o Highly recommended that a concrete or similar base be used to secure bottom of the fence and to prevent animals burrowing under fence.<br>o An SOP is required detailing when and who checks the fence. This must be done at least weekly and a register must be kept detailing any faults and corrective actions.<br>o No domestic animals shall be allowed inside the fenced area of a piggery.
01.	Adhere to ideal **pig stocking density recommendations**. Separate sick pigs from healthy pigs.<br>• The guidelines for the stocking density in pig stables are determined in the EU legislation (EU, 2008). However, these norms are based on outdated research as well as insights and have not evolved with the recent evolutions in the industry (Pork360, n.d.). Therefore, the values are not in line with the optimal guidelines

	and should be considered as absolute minimum requirements rather than the ideal values:<br>• Prepare a plan for the number of pens/crates, the area of pens/ size of crates, area per pen, maximum number of pigs per pen and the number of pigs that can be housed in each and every building.<br>• If pens of differing sizes are present in a house, the information on area, space allowed and max. animals/pen must be reflected for the different size pens<br>• The minimum floor space allowances for post-wean pigs shall be adhered to.<br>• Sows in group housing should have 2.25 m2 per sow (Pork 360)
32.	**Control of scavenger animals** (e.g. sacred ibis, feral cats, feral dogs, feral pigs)<br>• Control scavenger access to pig farm or around the farm (within 5 km of the compartment unit premises).<br>• Fence in compartments with non-porous/stock-proof fencing.<br>• Bury carcasses and organs to a proper depth (3 m) following slaughter.<br>• A comprehensive documented program (SOP) should be in place for the control and extermination of scavengers where necessary.<br>• A responsible person must be identified and trained as scavenger animal control officer.
18.	• Hunters should have **no contact with pigs within 48h** after hunting activity.<br>• Wild pig carcasses should be found and disposed of quickly. The disposal of carcasses to be carried out either by deep burial, bringing to the rendering plant or burning (under supervision of the competent veterinary authorities).<br>• Possible use of appropriate chemicals for local disinfection of the carcass burial site.
23.	Implementation of (minimum) **tick and fly control measures**<br>• Fly control in and within 5 km of the compartment premises where and if possible<br>• Implementation of insect control can include control of ticks, especially of *O. savignyi* (tampan) ticks, to reduce the risk of transmission of ASF virus from ticks to pigs

33.	**Thorough pest control** (e.g. mice, rats, birds) within the compartment unit premises<br>• A comprehensive documented program (SOP) should be in place for the control and extermination of vectors (rats/ mice/ flies / insects, feral cats) where necessary.<br>• A responsible person must be identified and trained as pest control officer.<br>• All pest control stations, if used, must be clearly indicated on the farm plan
12.	Enforcement of **boot cleaning & disinfection or boot & clothes changing** of external people, livestock field officers/ veterinarians/ para-veterinarians, and compartment personnel that are in contact with pigs upon entering the pig site<br>• Establish foot dips for disinfection at the entrances to the pig houses.<br>• Change rubber boots/slippers upon entering pig houses.<br>• Change clothing when going in/out of pig houses.<br>• Establish a biosecurity plan that is approved/recommended by veterinary services that includes physical cleaning, washing and drying, disinfecting, and rest period.<br>Enforcement of **cleaning & disinfection of facilities and equipment** of compartment personnel that are in contact with pigs<br>• Routine (regular) cleaning of facilities and equipment with effective disinfectants.<br>• Remove manure and litter from facilities and equipment routinely.<br>• Establish a biosecurity plan that is approved/recommended by veterinary services that includes physical cleaning, washing and drying, disinfecting, and rest period.
03.	Compartment units neighboring ASF-infected farms can implement a **provisional quarantine of the compartment units**<br>• Stop all animal movement (in and out) unless necessary for slaughtering<br>• Intensified movement restriction of personnel and service providers (i.e. feed supply, etc.).
25.	**Thorough decontamination of non-swine delivery vehicles** entering & leaving the compartment premises especially when also used for internal movement of pigs, feed or raw materials<br>• Washing and disinfection of vehicles.<br>• Designated area for washing and disinfection of vehicles.<br>• Vehicle Access restrictions and biosecurity.

	• Down time for vehicles.<br>• Use appropriate vehicle stocking densities.<br>• Biosecurity plan approved/recommended by veterinary services<br>For more details, see Pork360 guidelines 1.3 and 2.1-2.2
24.	**Thorough decontamination of swine transport vehicles** entering & leaving the compartment premises and **thorough decontamination of vehicles used to transport pigs** from the compartment to the abattoir between shipments especially when also used for internal movement of pigs, feed or raw materials<br>• Washing and disinfection of vehicles.<br>• Designated area for washing and disinfection of vehicles.<br>• Vehicle Access restrictions and biosecurity.<br>• Down time for vehicles.<br>• Use appropriate vehicle stocking densities.<br>• Biosecurity plan approved/recommended by veterinary services<br>For more details, see Pork360 guidelines 1.3 and 2.1-2.2
14.	• Develop capacity of compartment personnel and farm managers on ASF, especially on recognition of ASF clinical signs. See section 5.2 for more details.<br>• All deaths of post-weaning animals and adult animals must be recorded in a register indicating suspected reasons.<br>• Check the ASF reporting system through a simulation exercise for a suspect ASF case to identify and address gaps in the reporting system<br>• Raise awareness among compartment personnel in contact with pigs on the importance of reporting suspect cases to farm managers, veterinary and livestock field officers<br>• Build trust between compartment personnel and farm managers and between farm managers and veterinarians through risk communication.
