# Supplementary material for: Analysis of the drivers of ASF introduction into the officially approved pig compartments in South Africa and implications for the revision of biosecurity standards
Source: Porcine Health Manag. 2022 Oct 6;8:43. doi: 10.1186/s40813-022-00286-7 (PMC9540751; doi:10.1186/s40813-022-00286-7)
Supplement: Supplementary file 7 — Additional file 7. Rating of Uncertainty Scores in Expert Elicitation. [file 40813_2022_286_MOESM7_ESM.pdf]

Additional File 7 – Rating of Uncertainty Scores in Expert Elicitation

**Rating**	**Explanation**
1 Very confident	I am very confident in my opinion on the score of the category of risk factors because I have extensive knowledge or experience on the topic OR I am familiar with the available robust scientific research on the topic relating to the category of risk factors
2 Somewhat confident	I am somewhat confident in my opinion on the score of the category of risk factors because I have some knowledge or experience on the topic OR I am familiar with some of the available scientific research on the topic relating to the category of risk factors
3 Not at all confident	I am not at all confident in my opinion on the score of the category of risk factors because I have limited knowledge or experience on the topic, I am not familiar with the available scientific research on the topic OR there is limited available scientific research on the topic relating to the category of risk factors
